# Personal Trust Increases Cooperation beyond General Trust

**DOI:** 10.1371/journal.pone.0105559

**Published:** 2014-08-21

**Authors:** Cristina Acedo-Carmona, Antoni Gomila

**Affiliations:** Department of Psychology, University of the Balearic Islands, Palma de Mallorca, Spain; Center of nonlinear, China

## Abstract

In this paper we present a new methodology which, while allowing for anonymous interaction, it also makes possible to compare decisions of cooperating or defecting when playing games within a group, according to whether or not players personally trust each other. The design thus goes beyond standard approaches to the role of trust in fostering cooperation, which is restricted to general trust. It also allows considering the role of the topology of the social network involved may play in the level of cooperation found. The results of this work support the idea that personal trust promotes cooperation beyond the level of general trust. We also found that this effect carries over to the whole group, making it more cohesive, but that higher levels of cohesion rely on a particular topology. As a conclusion, we hypothesize that personal trust is a psychological mechanism evolved to make human social life possible in the small groups our ancestors lived in, and that this mechanism persists and plays a role in sustaining cooperation and social cohesion.

## Introduction

Current approaches to the evolution of cooperation share the notion that any viable account must assume that cooperation is in the interest of the cooperator. All these models take it for granted that agents are self-interested, and that the only way to account for the evolution of cooperation is to show that it is in the interest of the agents. This can be due to the fact that reciprocity can be beneficial to both parties, at different times, and can be reinforced either directly [1], or indirectly [2], [3]; or to the fact that defectors are somehow punished [4], [5], [6], [7]. Thus, a rational agent is expected to cooperate in an iterated prisoner's dilemma (IPD) just when it is in its own interest and in addition is able to resist the discount of the future.

This standard model of the rational agent, though, does not fare well with the evidence, which rather reveals the existence of genuine pro-social preferences in humans: an interest in another's welfare, even if this may involve a cost to oneself [8]. Therefore, these social preferences should be included in the explanation of the evolution of cooperation [9], [10], [11]. This requires providing an account of how these social preferences evolved in the first place, and how they sustain the forms of cooperation that can be found across societies. However, social preferences are still regarded with skepticism by some researchers [12], and have not yet found a proper treatment in evolutionary games models. In part, this is due to the conditional strategies followed by agents, which may cooperate or defect depending on the partner's decisions. This conditionality may be interpreted as suggesting that agents are not guided by social preferences in general, always and everywhere, which may also invite a self-interested account –social preferences as a form of hypocrisy.

In this paper, we want to contribute to the defense of social preferences by focusing on personal trust, a powerful psychological mechanism that can be seen, from an evolutionary point of view, as a way to solve social dilemmas by making one feel certain that our counterpart will be loyal and choose to cooperate and hence, making one feel committed to cooperate. This is achieved, neither by an external threat of punishment, nor by some sort of rule-enforcing authority, but by an affectively grounded, benevolent attitude towards the trusted person that is derived from previous interactions [13], [14]. In other words, personal trust puts one in a situation of risk of being exploited, while believing that one will not be exploited, because of the feeling that binds one with the counterpart. Therefore, it is a complex psychological state, which relates two people, with a previous story of positive interactions, and which involves both a cognitive and an affective dimension, and which gives rise to a pro-social attitude between them.

Many proposals have underlined the role of trust in our social life. Trust is of great interest in the social [15], [16], political [17], [18], [19], [20], and economic sciences [21], [22], [23], that consider cooperation to be a fundamental aspect of the organization and maintenance of cohesion in the large societies of nowadays. These disciplines are interested in which factors increase the level of trust and cooperation within and between societies. The factors that have received more attention are the creation of rules, institutions, ideologies, and the promotion of social habits that increase and support social networking for practical purposes –under the notion of “social capital” [24], [25], [26], [27]. Similarly, social psychology has explored the differences between intra- and inter-group behavior, as regards cooperative behavior [28], [29].

It's arguable, however, that these approaches have mostly relied on the notion of general, rather than personal trust [30], [27], [31], [32], [33]. General trust is an attitude towards any other person. It is clear that this general attitude fosters cooperation [34] and provides conditions concerning communication, reputation, etc. [35]. As a matter of fact, general trust can be viewed as the psychological factor in play in so-called “trust games” in experimental economics, where “trust” is just taken to mean an expectation about how the partner will choose [36], [37]. General trust can help explain the robust fact that in games played anonymously, cooperation is found about 50% of the times in the first round and then decays [38], [39], [40], [23]. But typical game playing is incompatible with personal trust because it is designed to keep anonymity. Therefore, even though general trust may contribute to cooperation, what remains to be shown is whether personal trust may also have a role -one that may be even more important [41], [42].

In fact, personal trust can help explain the pro-social attitudes observed in many cooperative behaviors. It proceeds through the tendency, often unconscious, to cooperate more with those people one trusts, because this affective bond involves an implicit expectation of reciprocity. In addition, an evolutionary perspective clearly suggests that human social life finds its roots in small groups, where everybody can interact with everybody else, and is known by everybody in the group. This suggests that it is personal trust that matters in these small-scale groups [43]. Even if it were possible to develop global measures of social cohesion (such as social capital theory suggests), the particular bonding pattern of particular members –as suggested by personal trust–, seems to be the central factor in fostering cooperation, the one upon which any other relies, even in large societies.

Interestingly, recent work on evolutionary games provides indirect theoretical support to this approach. Several models introduce as a new factor some sort of heterogeneity among the individuals, so that individuals in a social network are not equally likely to interact with each other [44], [45], [46]. Personal trust may be “the missing link” in these models, the psychological mechanism by which the network topology is structured. The critical point is that the network topology fosters cooperation by itself. From this point of view, this theoretical work can be interpreted as a way to account for the evolution of social preferences. Our empirical study will also consider this implication.

Therefore, we hypothesize firstly that personal trust has a greater weight than general trust in fostering cooperation (H1). Furthermore, we also hypothesize that personal trust among group members is the key to the cohesion of the social group, so that the higher the level of personal trust, the greater the group cohesion (H2). In other words, personal trust fuels cooperation even with non-personally trusted agents, just because it structures social networks. Consequently, we also hypothesize that personal trust generates a characteristic social network of cliques, which is the structure through which cooperation spreads beyond the trust circle (H3).

To test these hypotheses, this work integrates several methodologies: questionnaires, an experimental game –an iterated prisoner's dilemma–, and social network analysis. The questionnaires allow us to measure both general and personal trust, in a group of people. The experimental game allows us to organize participants in such a way that, while keeping anonymity, they can play with one of their trusted members within the group. It also allows, by letting the participants know that it will be played three times, to check whether cooperation is conditional on strategic calculation (of reciprocity or backwards induction), or it is based on personal trust. We also pay attention to the social network structure [47], [48], [49], [50], [51], [52], [53], [54] and cooperative behaviors [55], to ascertain whether social network topology has a role of its own in fostering cooperation [56], [57].

This study builds on a previous pilot study [58], which already showed an effect of personal trust on cooperation. This work tries to overcome the limitations of the previous one, regarding the number of participants and the time of previous interaction within the group. We have also improved our questionnaires to measure general and particular trust. In addition, we have developed an in-depth analysis of the structure of the trust networks involved.

## Methods

### 1. Ethics statement

This study was approved by the Ethics Committee of the University of the Balearic Islands. Writen informed consent was obtained from each participant prior to participation, as approved by the Ethics Committee.

### 2. Participants

Participants in this study were two groups of 54 third-year undergraduate students: a group of 40 Psychology (PSYCHO) students, and a group of 14 Physiotherapy (PHYSIO) students, from the University of the Balearic Islands. Their global characteristics are: 26% males and 74% females; aged between 20 and 49 years old (Mean± SE = 22.43±0.67, N = 54); mostly of Spanish nationality; 41% Catholics, 57% declared non-believers, and 2% Orthodox; in their vast majority just students −80%–, while the rest carry out other activities: 9% part-time employees, 2% liberal professionals and 2% government workers; 93% are unmarried. About their economic possibilities, 80% has monthly expenses below 500 euros, 15% are consumers of between 500 and 1,000 monthly euros and 5% has expenses of more than 1,000 monthly.

### 3. Procedure

#### 3.1. Trust measures through questionnaires

Different scales have been developed to measure trust, aimed at different goals [59], [60], [61], [62], [63], [64], [65], [66], including occasionally the analysis of close relationships [67]. For our study, we have chosen the most well-established items in the literature to prepare our own questionnaires: one for general trust and one for personal trust. Whereas personal and general trusts are different notions, they are somehow related; that's why some items are repeated in both questionnaires.

The general trust questionnaire (see Annex S1) involves 5 questions widely used in literature concerning attitudes towards other people in general: one on perceived fairness [68]; one on relational trust [33], and three questions on what trust is about: money, secret information, and care of beloved ones [61], with answers ranging on a 5-point Likert scale.

To obtain a measure of personal trust, we first asked our participants to name 5 people they trusted in their classroom group, and then they had to answer our second questionnaire, about particular trust, for 3 of them (see Annex S2). Six questions concern the participants' expectations about their trustees on lending and borrowing money, caring for the beloved ones and sharing secrets –similar to some questions used in the general trust questionnaire, but now related to the particular individuals they say they trust–, and two more questions to get one more accurate measure of the level of personal trust according to each particular trustee such as: getting help if moving, or being defended by the trustees at their own expense or personal effort. All of them are to be rated on a 5-point Likert scale as well.

Each measure is then expressed as a percentage, with 100% meaning maximal trust (either general or personal).

#### 3.2. Iterated prisoner's dilemma

Several days after filling in the questionnaires, each participant played an iterated prisoner's dilemma, with 3 repeated decisions, paired with another member of the group, in two experimental conditions. In one condition –trust circle condition (TC)–, each participant played with somebody from their trust circle without knowing which one in particular. In the other condition –non-trust circle condition (NTC)–, the iterated prisoner's dilemma was played anonymously with somebody also from the classroom, but not mentioned as a trusted one. Players were placed in different rooms, and their respective decisions were communicated after each round of the game. Half the participants played first in the TC, half played first in the NTC. Both participants had the same information and were under the same conditions.

**Figure 1:**
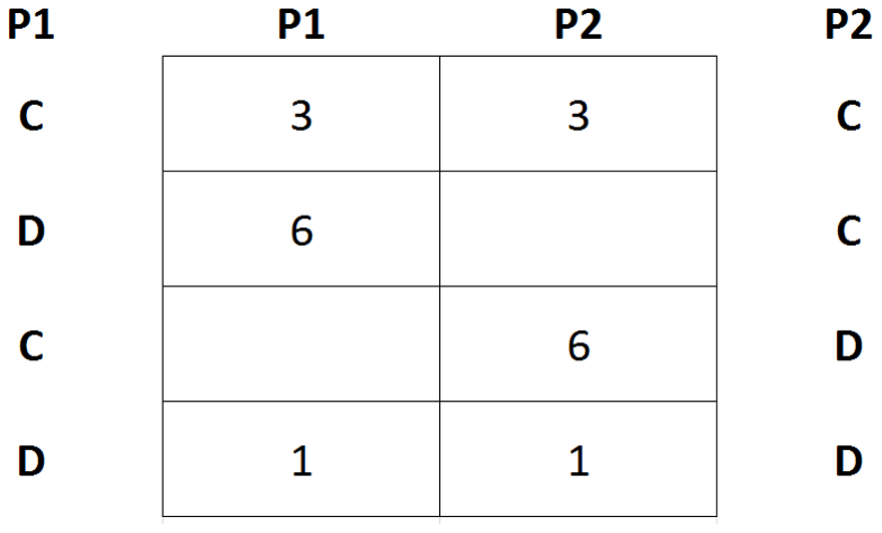
The prisoner's dilemma pay-off matrix: (C) means cooperate and (D) means defect, (P1) is participant 1 and (P2) is participant 2.

In each round, participants were asked to decide whether cooperate with, or defect to, their partner, before knowing the other player's decision, in order to obtain a number of points which depended on the decisions of both players, with the possibility of obtaining a maximum of 6 points or a minimum of 0 points in a round, as specified in the pay-off table (Fig. 1). Most importantly, they knew in advance they would be playing the game three times in each condition, as the way to tell apart self-interested cooperation from trust-inspired cooperation. The decline in cooperation as the game proceeds is only expected when cooperation is driven by self-interest [38], [39], [40].

According to the number of points obtained after going through both conditions, participants got different awards. The higher the score, the bigger the prize won. The prizes ranged from a ticket for a snack to a pack of CDs. However, participants were ignorant of the prizes until all of them completed the study –in order to makethem play under the same conditions.

## Results

### 1. Particular and general questionnaires

**Figure 2:**
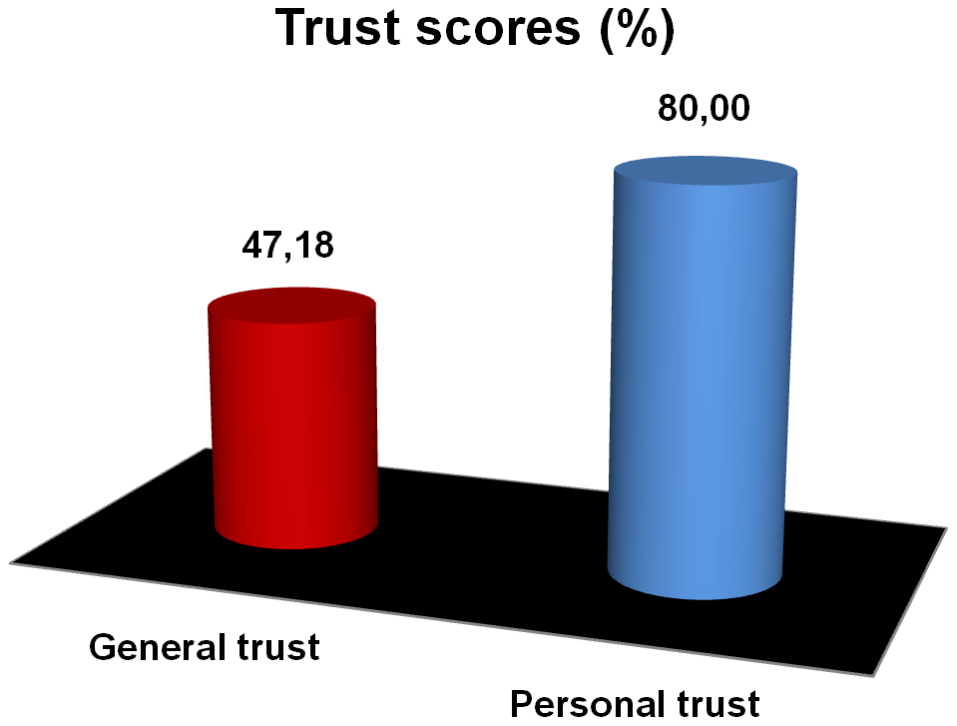
Global scores of general and personal trust.

Global personal and general trust scores are represented in Fig. 2. Personal trust scores are higher than general trust scores.

We obtained a significant difference –Wilcoxon text– (z = −6.393, N = 54, p<0.001, r = −0.86) between means of personal trust scores (Mean± SE = 7.99±0.13, SD = 0.96, N = 54) and general trust scores (Mean± SE = 4.71±0.12, SD = 0.91, N = 54).

**Figure 3:**
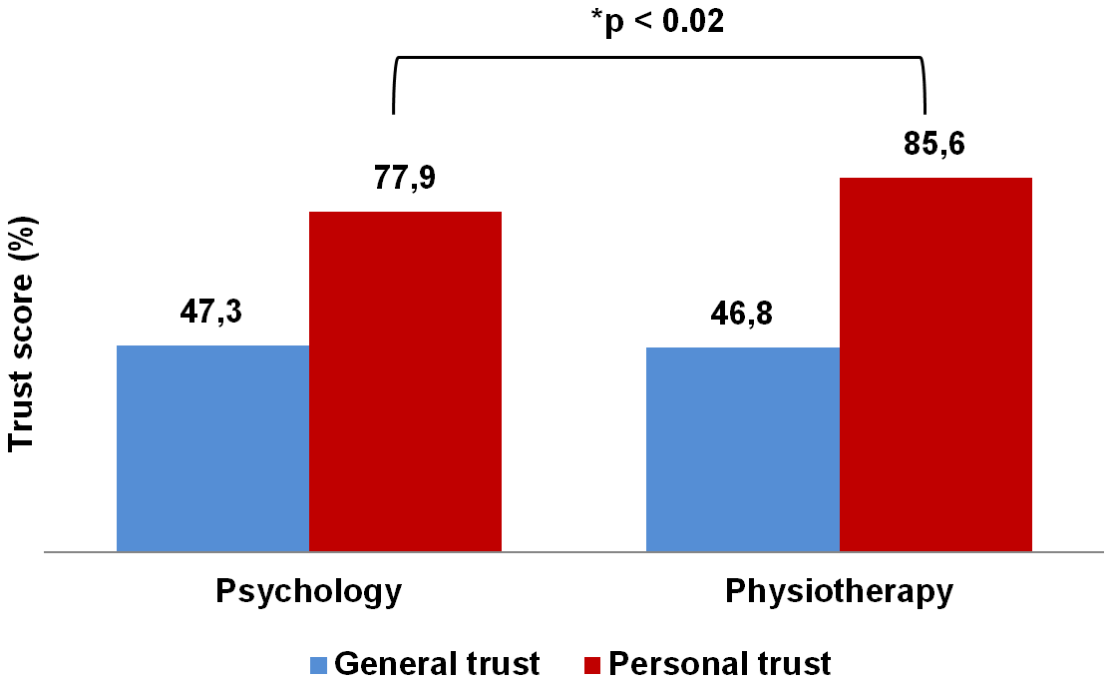
General and personal questionnaires scores by groups. (*) Significant difference in personal trust score between PSYCHO and PHYSIO groups –Mann-Whitney test.

Personal and general trust scores were also calculated separately for the PSYCHO (General trust score: Mean ± SE = 46.8±1.80, SD = 6.73, N = 14; Personal trust score: Mean ± SE = 85.6±2.60, SD = 9.73, N = 14) and PHYSIO (General trust score: Mean ± SE = 47.3±1.56, SD = 9.87, N = 40; Personal trust score: Mean ± SE = 77.9±1.40, SD = 8.85, N = 40) groups. We found that, while the differences in general trust score were not significant –Mann-Whitney test– (U = 270.5, z = −0.19, N = 54, p<0.9, r = −0.02), the differences in personal trust score were significant (U = 158, z = −2.41, N = 54, p<0.02, r = −0.32) (Fig. 3).

Detailed means and standard deviations obtained for each item of the questionnaires appears in Tab. 1 for general trust and Tab. 2 for personal trust.

**Table 1:** Results of the general trust questionnaire by items and groups.

Scale and items	Mean (SD)	PSYCH mean (SD)	PHYSIO mean (SD)
General trust scale	2.36 (0.60)	2.38 (0.61)	2.34 (0.61)
Do you think most people would try to take advantage of you if the got a chance or would rather try to be fair?	3.24 (0.97)	3.20 (1.01)	3.35 (0.84)
Generally a person with whom you have had a longer relationship is likely to help you when you need i	2.22 (1.16)	2.27 (1.26)	2.07 (0.82)
Would you lend some of your property to an unknown person?	2.50 (1.01)	2.51 (1.04)	2.50 (0.94)
Would you leave the care of someone important to you to a stranger person?	1.57 (0.71)	1.47 (0.64)	1.85 (0.86)
Would you share personal information with stranger people?	2.29 (0.86)	2.42 (0.84)	1.92 (0.82)

**Table 2:** Results of the personal trust questionnaire by items and groups.

Scale and items	Mean (SD)	PSYCH mean (SD)	PHYSIO mean (SD)
Personal trust scale	4.00 (0.40)	3.89 (0.41)	4.28 (0.36)
Do you think that XX would lend you a large sum of money if he/she had so much?	3.71 (1.00)	3.59 (1.03)	4.07 (0.83)
Do you think that XX would pay back to you a loan of a large sum of money?	4.50 (0.75)	4.43 (0.74)	4.71 (0.74)
Would you leave to XX the care of something very valuable to you?	4.30 (0.80)	4.17 (0.85)	4.66 (0.52)
If there was a secret that would be very damaging to you were it to become public, would you share it with XX?	3.85 (1.09)	3.71 (1.13)	4.23 (0.87)
Do you think XX would help you if you were to move?	4.18 (0.94)	4.15 (0.91)	4.26 (1.03)
If to defend you XX could get injured, do you think he/she would do so?	3.43 (0.89)	3.33 (0.90)	3.73 (0.79)

Personal and general trust scores may be partially connected [69], although they are different measures. In fact, we found that personal trust score correlates –one-tailed Kendall's tau measure– with general trust score (r = 0.256, N = 54, p<0.01).

### 2. Cooperative behavior in prisoner's dilemma

**Figure 4:**
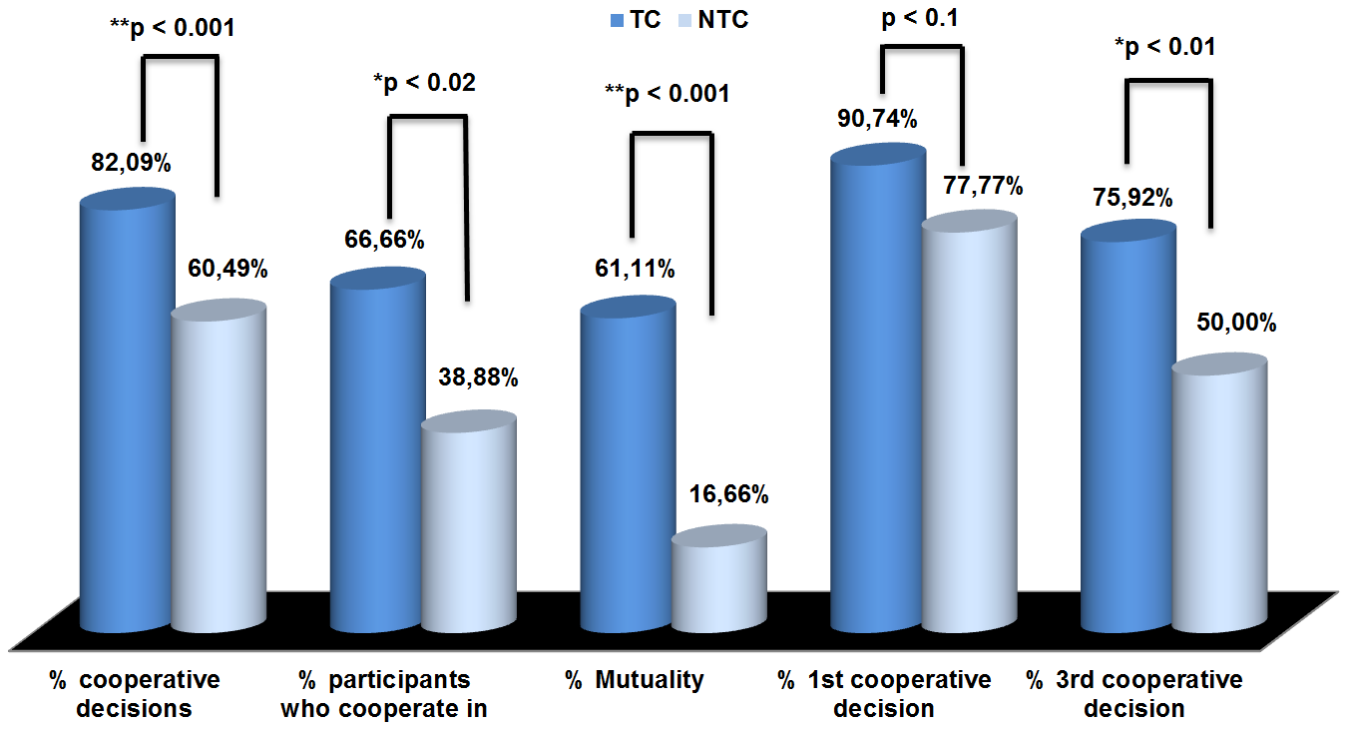
Level of cooperation in the IPD. (*) Significant and (**) very significant differences –One-tailed McNemar tests.

To measure the level of cooperation, we developed four indicators: a) proportion of cooperative choices; b) proportion of participants who cooperate in the 3 decisions; c) proportion of mutuality, i.e., when both players always cooperate; and, d) the contrast between the first and in the third rounds. We compared these data in both conditions. Results appear in Fig.4.

One-tailed McNemar tests demonstrated that there was a significant higher proportion of cooperation for each of the dependent variables in the TC than in the NTC, except as regards the first decision, whose differences failed to reach significance. Thus, the percentage of cooperation was 82.09% in the TC, and 60.49% in the NTC (X2 (1, n = 54) = 11, p<0.001); the percentage of participants who always cooperate was 66.66% in the TC and 38.88% in the NTC (X2 (1, n = 54) = 6.53, p<0.02); the percentage of mutuality was 61.11% in the TC and 16.66% in the NTC (X2 (1, n = 54) = 16.94, p<0.001); and in the TC cooperation shifted from 90.74% in the first round to 75.92% in the third one, while in the NTC, cooperation shifted from 77.77% to 50.00%: for the first round, (X2 (1, n = 54) = 3.26, p<0.10); for the third round, (X2 (1, n = 54) = 7, p<0.01).

We also found the general tendency to defect on the final round, but the decline in cooperation from the first to the final decision was higher in the NTC (X2 (1, n = 54) = 9.03, p<0.01) than in the TC (X2 (1, n = 54) = 4.26, p<0.05), according to a Pearson chi-square measure.

### 3. Analysis of trust networks

Given the significant differences found in personal trust between the two groups, we decided to analyze whether they were related to differences in group cohesion, as hypothesized (H2). We built the trust networks of the two groups –PSYCHO and PHYSIO–, on the grounds of the group members info supplied in the personal trust questionnaire by each participant, using the Gephi software [70]. Given the different number of participants in each group, we also compared the structure of each network to an equivalent random one (one of identical number of nodes and links), which represents the null hypothesis. In this way, we could also examine our hypothesis concerning the role of network topology to cooperation (H3).

**Figure 5:**
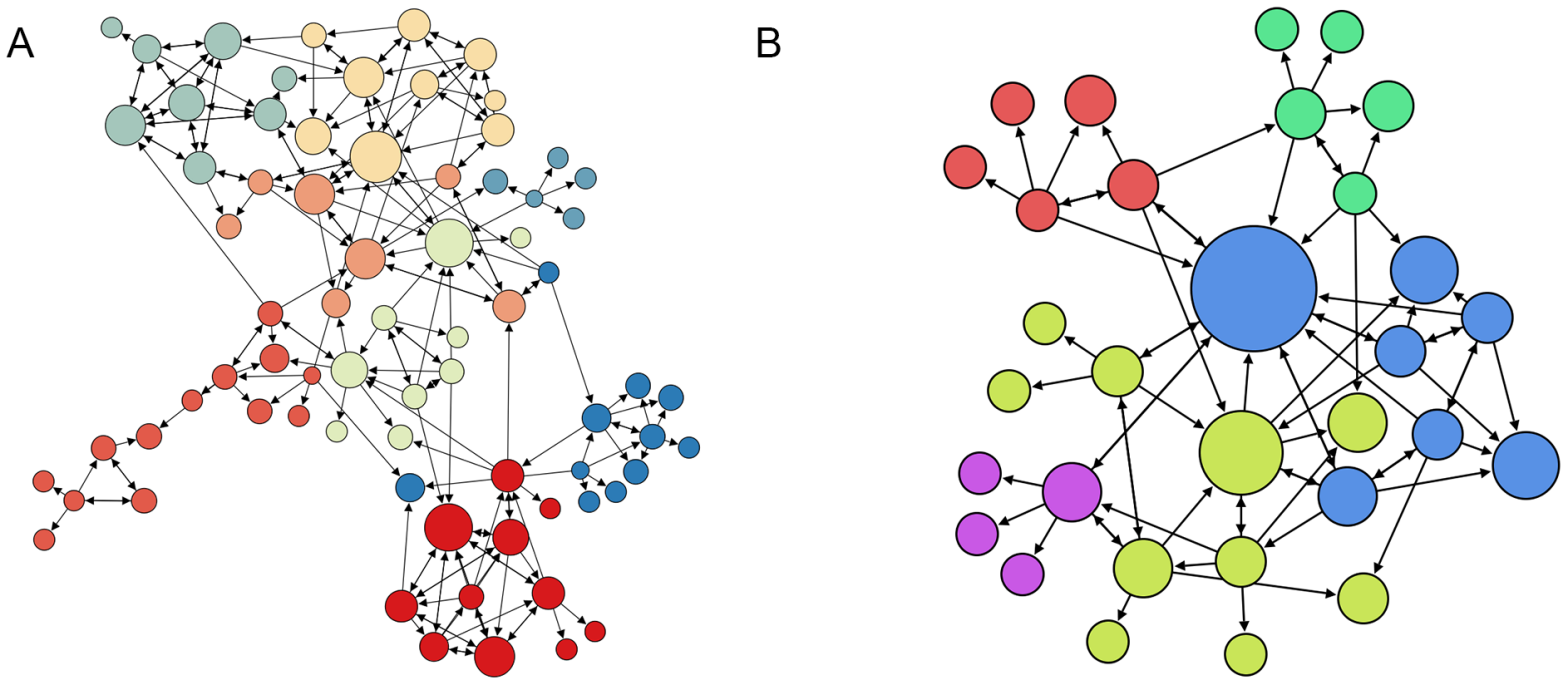
The PSYCHO trust network (A) and the PHYSIO trust network (B). The nodal size represents the in-degree level and the nodes colors represent the communities –modularity.

The network structure of the 2 groups –PSYCHO and PHYSIO– appears in Fig. 5. Each participant and each mentioned trustee are represented as nodes, while links represent the trust relationships; therefore, they represent who trusts whom in the group. The size of the node indicates how many times a participant was mentioned as someone trusted by the other members of the group –in-degree level.

Several network measures were usedto determine the level of network cohesion:

- Clustering coefficient [71], [72]: indicates how the nodes are embedded between its neighboring nodes. The average gives a general indication of the clustering into the network.
- Modularity [73]: it is a detection algorithm of communities. A result of 0.4 or greater value is considered generally significant. Social cohesion runs against modularity.
- % of reciprocity: percentage of mutual edges with respect to the total edges of the network, that is, couples that name each other as trustees in the personal trust questionnaire. The greater reciprocity in a group, the less cohesive it is because when the number of people to trust is smaller –a less cohesive group–, people tend to rely on mutuality.
- Average path length [74]: graph average distance between all pairs of nodes. Connected nodes have distance 1. A shorter average path length indicates greater cohesion of the network.
- Diameter [74]: it is the longest graph distance between any 2 nodes of the network –how far are the 2 nodes further away. The meaning of this measure is very similar to the previous one.

**Figure 6:**
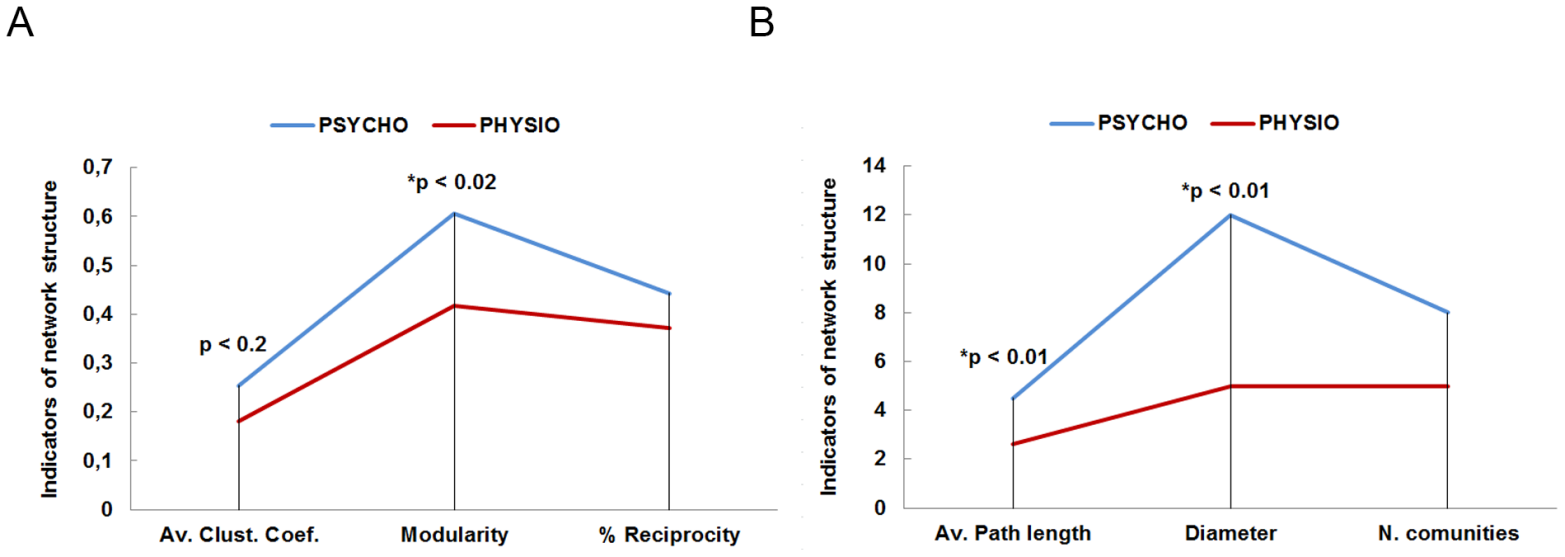
It shows the comparison of the indicators of network structure between the PSYCHO (A) and PHYSIO (B) groups. (*) Significant differences respect to their equivalent individual measures (clustering, modularity, closeness, eccentricity) –Man-Whitney tests.

Fig. 6 represents the results for each group. We found that in the PSYCHO group there were more, and more intensively related, internal communities –as indicated by the clustering coefficient and modularity– than in the PHYSIO group. In addition, the distances among nodes –average path length and diameter– are also longer in the PSYCHO group than in the PHYSIO group. These results indicate a higher level of cohesion for the PHYSIO than in the PSYCHO network.

To examine if these differences between the structures of the two networks are significant, we proceeded to an individual analysis on the set of nodes of the networks. In this case, we looked at measures equivalent to the above ones at an individual level: clustering coefficient; modularity; and average path length and diameter that can be measured at an individual level by closeness centrality and eccentricity respectively. Thus, closeness centrality refers to the average distance from an initial node to all other nodes in the network, and eccentricity refers to the distance from a node to the most far off one from it in the network. A Mann-Whitney test found significant differences between PSYCHO and PHYSIO network structure in modularity (U = 780.5, z = −2.521, N = 104, p<0.02, r = −0.24), eccentricity (U = 740, z = −2.935, N = 104, p<0.01, r = −0.28), and closeness centrality (U = 739.5, z = −2.934, N = 104, p<0.01, r = −0.28) but not in clustering coefficient (U = 935.5, z = −1.421, N = 104, p<0.2, r = −0.13).

**Figure 7:**
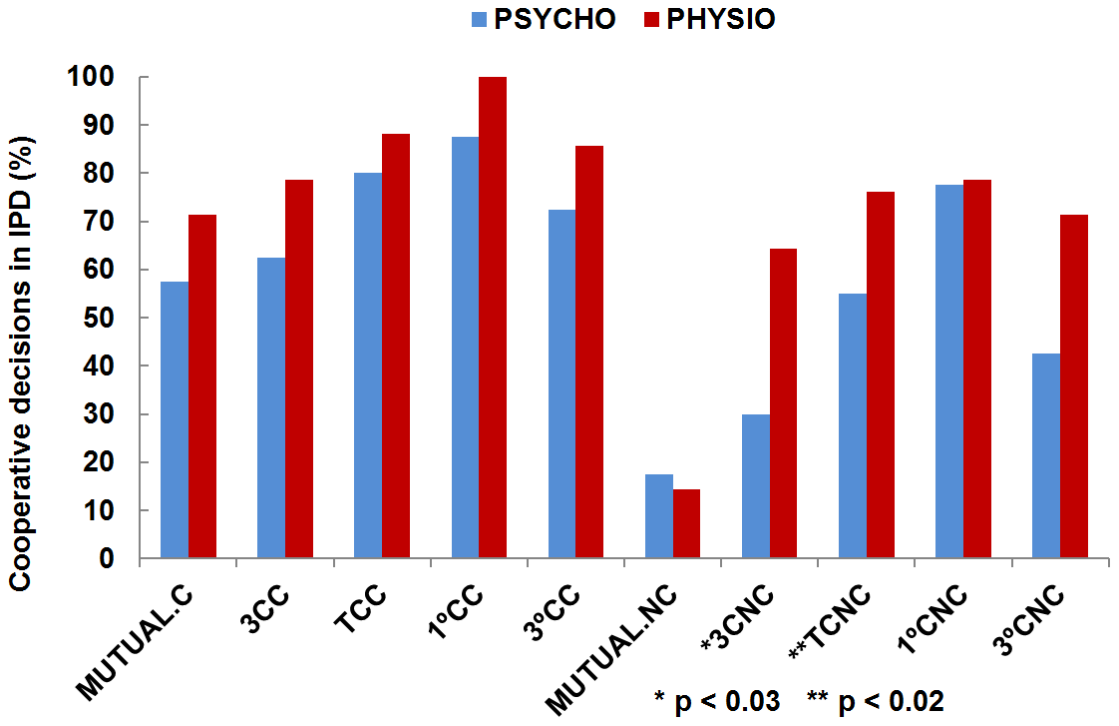
Cooperation results in the Prisoner's Dilemma by groups. MUTUAL refers to the proportion of mutuality, when both players always cooperate; 3C refers to the proportion of participants who cooperate in the 3 decisions; TC refers to the total proportion of cooperative decisions; and 1°C and 3°C refers to the contrast between the cooperative decisions in the first and in the third rounds. The following C or NC refers to trust circle or non-trust circle conditions. (*) (**) Significant differences –Pearson Chi-square tests.

On the other hand, the results of cooperation in the iterated prisoner's dilemma for each group separately exhibited non-significant differences in the TC by means ofPearson Chi-square tests: mutuality (X2(1, n = 54) = 0.84, p<0.4); cooperation in the three decisions (X2(1, n = 54) = 1.20, p<0.3); total of cooperative decisions (X2(1, n = 54) = 1.38, p<0.3); cooperation in the first decision (X2(1, n = 54) = 1.92, p<0.2); cooperation in the third decision (X2(1, n = 54) = 0.991, p<0.4); and barely significant differences in some measures of cooperation in the NTC, such as cooperation in the three decisions (X2(1, n = 54) = 5.129, p<0.03); and total of cooperative decisions (X2(1, n = 54) = 5.84, p<0.02). The other measures in the NTC –mutuality (X2(1, n = 54) = 0.07, p<0.8); cooperation in the first decision (X2(1, n = 54) = 0.007, p<0.95); and cooperation in the third decision (X2(1, n = 54) = 3.47, p<0.07) showed non-significant differences (Fig.7).

**Figure 8:**
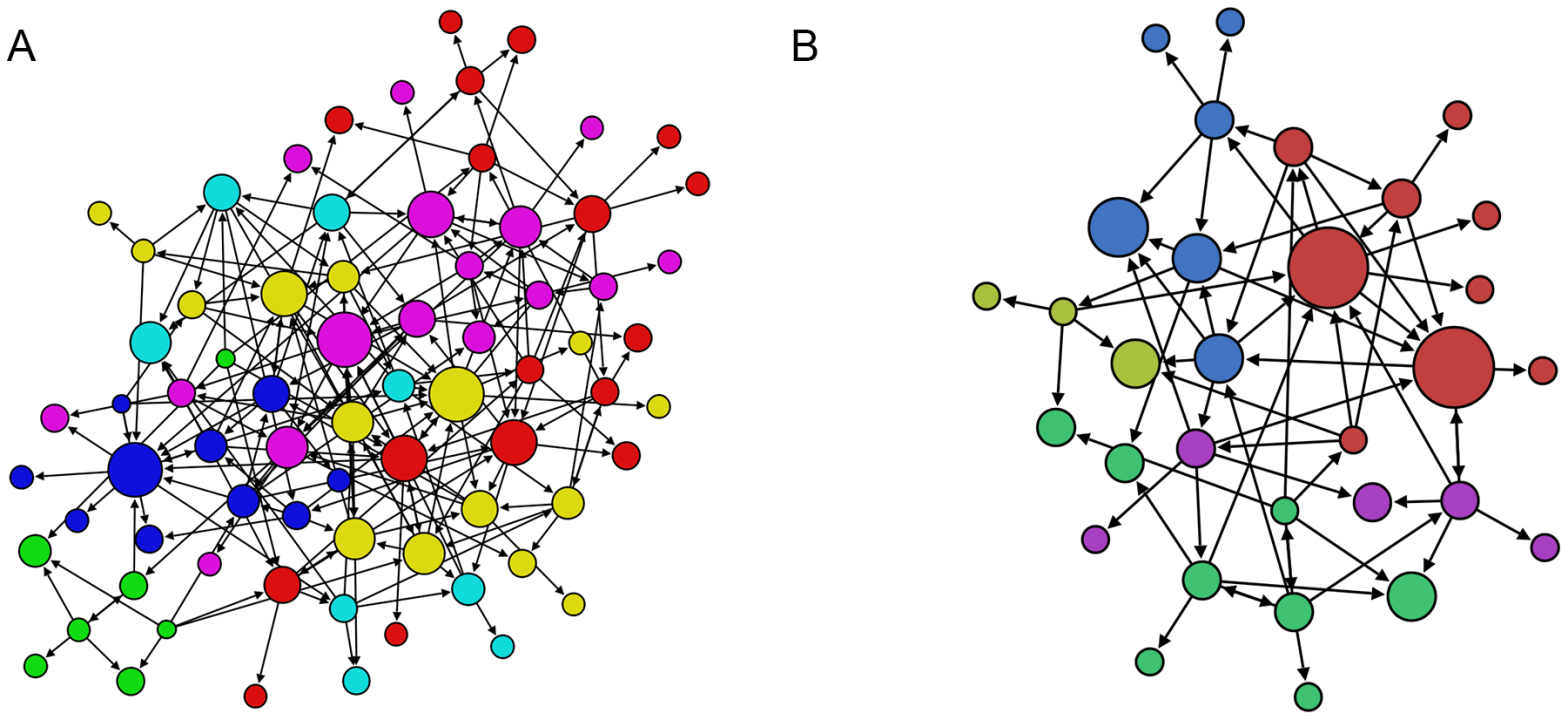
The random trust network of PSYCHO (A) and the random trust network of PHYSIO (B). The nodes size represents the in-degree level and the nodes color represents the communities –modularity.

In order to take into account the different sizes of these networks, we also compared them to their respective random networks [75], that is to say, networks with the same number of nodes and links but randomly connected (Fig.8).

**Figure 9:**
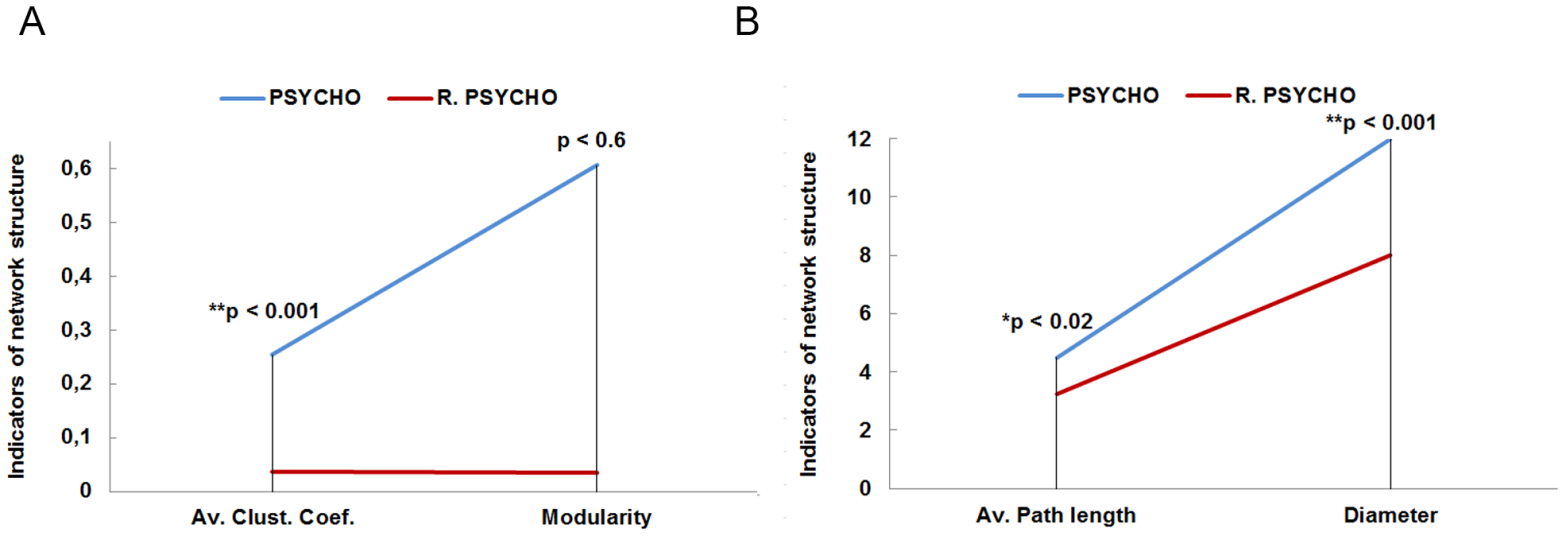
It shows the comparison of network structure indicators between the real (A) and random (B) trust networks of PSYCHO group. (*) Significant and (**) very significant differences respect to their equivalent individual measures (clustering, modularity, closeness, eccentricity) – Man-Whitney tests.

**Figure 10:**
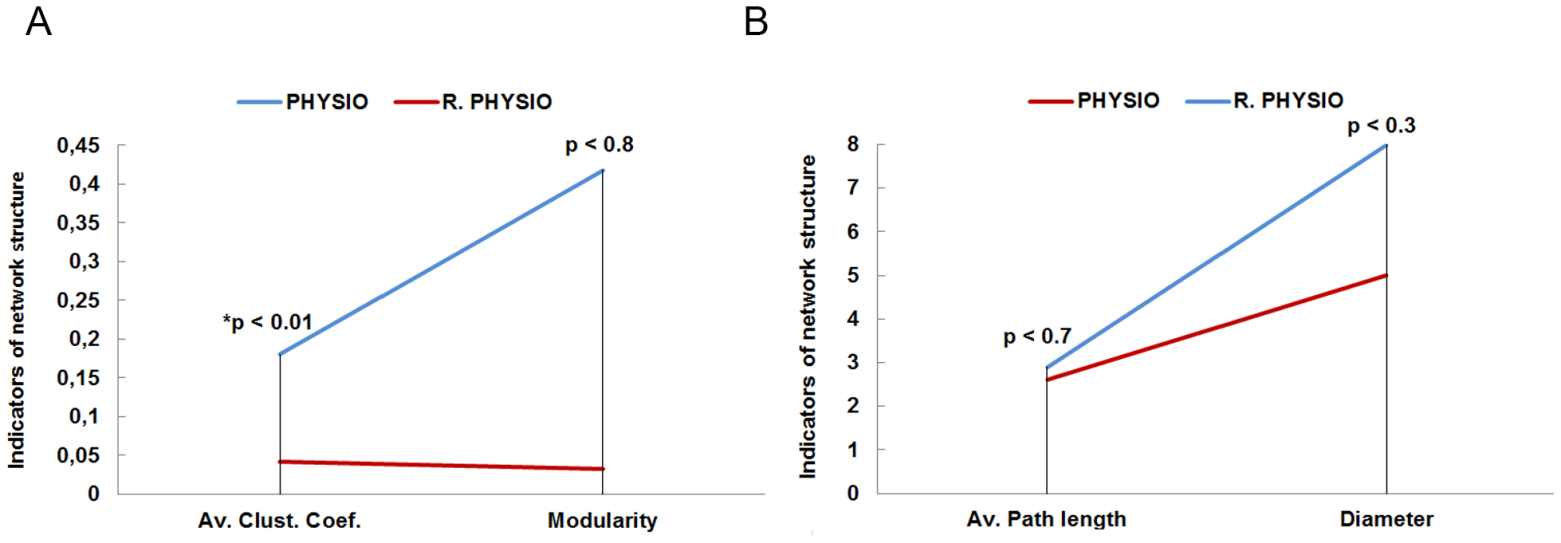
It shows the comparison of network structure indicators between the real (A) and random (B) trust networks of PHYSIO group. (*) Significant difference respect to their equivalent individual measures (clustering, modularity, closeness, eccentricity) –Man-Whitney tests.

Network measures comparing real and random trust networks are shown in Figure 9 for PSYCHO, and Figure 10 for PHYSIO networks. Significant differences between the PSYCHO network and its corresponding random network, both in their clustering coefficient and in the measures of distance between nodes at the individual level –eccentricity and closeness–, were found using the Mann-Whitney test: clustering coefficient (U = 1375.5, z = −5.25, N = 146, p<0.001, r = −0.43); eccentricity (U2042.5, z = −5.25, N = 146, p<0.001, r = −0.20); closeness (U = 2042.5, z = −2.53, N = 146, p<0.02, r = −0.20).Differences in modularity were not significant (U = 2513.5, z = −0.59, N = 146, p<0.6, r = −0.04).

Regarding the measures of PHYSIO and its random counterpart, differences in their clustering coefficients were significant, but differences in the distances among nodes and in modularity were not (Mann-Whitney test): clustering coefficient (U = 293, z = −2.84, N = 62, p<0.01, r = −0.36); eccentricity (U = 401.5, z = −1.22, N = 62, p<0.3, r = −0.15); closeness (U = 447.5, z = −0.50, N = 62, p<0.7, r = −0.06); and modularity (U = 454.5, z = −0.37, N = 62, p<0.8, r = −0.04).

## Discussion and Conclusions

This study, with bigger groups and longer-lasting relationships among their members than those of our previous study [58], provides robust support to the hypothesis that personal trust boosts higher levels of cooperation than general trust (H1). The highly significant differences found in cooperation between the two experimental conditions –trust and non-trust circle conditions– clearly show that personal trust increases cooperation beyond the baseline level commonly found even in one-shot anonymously played games. The most significant difference between the two experimental conditions was found in mutuality, which is in turn the most demanding measure of cooperation –it requires both participants to cooperate with each other in the three rounds. The fact that 75.92% of the decisions in the third round were cooperative in the TC, despite it was known by players that it was the last round of the game, clearly indicates that cooperation is driven by pro-social preferences, derived from personal trust, rather than by strategic calculation. The only non-significant difference found between the two conditions concerns the total amount of cooperative behaviors in the first decision, which may be attributed to the effect of general trust, and is coherent with the trend found in experimental games. In both conditions, cooperation in the first round is very high (90.74% in the TC and 77.77% in the NTC).

Our results also confirm that the way personal and general trusts were measured is well-grounded, even if there may be multiple ways to operationalize these notions. The average personal trust score (80%) –higher than the general trust score (47%)–, seems to be the critical factor giving rise to the high level of cooperation in social dilemmas found when the game is played anonymously among members of a trust circle, even if both measures are somehow correlated.

We also found support for our second hypothesis: that personal trust is the key to group cohesion, thus fostering cooperation even with non-members of the trust circle. Remember that the personal trust score is the group average of the trust level for each trust circle in the group. A group with a high level of personal trust will therefore foster higher levels of cooperation for the whole group: the fact that some people in the group are highly trusted by other members facilitates cooperation at the global level, thus reinforcing the cohesion of the network [46]. Support for H2 also comes from the comparative analyses of the networks of the PSYCHO and PHYSIO groups. There we found a significantly higher level of personal trust in the latter than in the former, which is congruent with the consistently higher scores for any of the measures of group cohesion. Remarkably, as expected, we found significant differences between both groups in total cooperation in the NTC: group cohesion is made apparent in that cooperation is easier with people outside the trust circle. Aware that the small and different size of both groups is a weakness, we compared each of them to a corresponding random network –with same number of nodes and edges–, as the way to clearly show that group cohesion has to do with personal trust.

Furthermore, comparative network analysis also makes possible to find evidence for our third hypothesis, that trust-based cooperation is better supported by a specific network topology: that of a small-world [72], in which the hubs correspond to cliques [76]. Several results support this idea: on the one hand, the structure of the PHYSIO network clearly exhibits this topology, while the PSYCHO network is not so well integrated. Consequently, the percentage of reciprocity –of pairs of participants that name each other as trustees– is higher in the PSYCHO than in the PHYSIO network (Fig.5), which is in line with the lower degree of cohesion of the PSYCHO network.

If this interpretation is correct, it calls for a modification of the “social circles” hypothesis [77], [78], [79], which distinguishes three kinds of social networks: the “support groups” – more or less 5 persons –, the “group of sympathy” –between 12 and 15 persons–, and the groups of other people with whom individuals establish sporadic relationships. The “social circles” hypothesis overlooks the potential of personal trust among non-kin to bind people together, in any sphere of activity of an individual. Similarly, network experiments that ignore the role of trust in social relationships do not find that network topology matters for cooperation [57], [80] –a result that indirectly suggests that trust-based network topology it's the factor that makes the difference. In fact, it seems to us that personal trust may be a central factor is giving rise to heterogeneity in social interactions, and therefore, in giving rise to a topology that may foster cooperation by itself [56], [46], [81], [82].

In summary, our multi-method study supports the view that personal trust is a crucial factor in cementing society. It is not the only factor: there may be other ways to foster cooperation and to make groups cohesive. However, personal trust creates robust and long-lasting bonds, which give rise to greater levels of social cohesion. We contend that these findings provide support for the view that small, trust-based groups are a basic social structure in non-kin groups. They were the evolutionarily original forms of social organization and can still be found across human societies, even in the large, developed ones. Personal trust drives psychological altruism towards non-kin, in a way that goes beyond the effect of general trust. It turns people into a kind of conditional cooperator: depending, not on whether there is the possibility to punish her if she defects; not on an authority that can require norm following; not on a strategic calculation about chances of reciprocation; but on an affectively mediated commitment derived from past experience of interaction. Further support for this view requires a cross-cultural anthropological approach.

## Supporting Information

Annex S1 **General trust questionnaire.**
(ODT)

Annex S2 Annex 2. Personal trust questionnaire
(ODT)
