# Supplementary material for: Personal Trust Increases Cooperation beyond General Trust
Source: PLoS One. 2014 Aug 21;9(8):e105559. doi: 10.1371/journal.pone.0105559 (PMC4140793; doi:10.1371/journal.pone.0105559)
Supplement: Supplementary file 1 [file pone.0105559.s001.odt]

Indicate with a cross on a scale of 1 to 5 the degree of your response to the following questions:

		**Take advantage**				**Be fair**
**1**	**Do you think most people would try to take advantage of you if they got a chance or would try to be fair?**	1	2	3	4	5
		a	b	c	d	e
		**Total Agree**				**Total Disagre**
**2**	**Generally, a person with whom you have had a longer relationship is likely to help you when you need it.**	1	2	3	4	5
		a	b	c	d	e
		**Sure not**				**Sure yes**
**3**	**Would you lend some of your property to an unknown person?**	**1**	**2**	**3**	**4**	**5**
		a	b	c	d	e
		**Sure not**				**Sure yes**
**4**	**Would you leave the care of someone important to you to a stranger person?**	**1**	**2**	**3**	**4**	**5**
		a	b	c	d	e
		**Sure not**				**Sure yes**
**5**	**Would you share personal information with stranger people?**	**1**	**2**	**3**	**4**	**5**
		a	b	c	d	e
