# Supplementary material for: Personal Trust Increases Cooperation beyond General Trust
Source: PLoS One. 2014 Aug 21;9(8):e105559. doi: 10.1371/journal.pone.0105559 (PMC4140793; doi:10.1371/journal.pone.0105559)
Supplement: Supplementary file 2 [file pone.0105559.s002.odt]

For each of the people mentioned above (XX), please answer to the following questions:

Indicate with a cross on a scale of 1 to 5 the answer to the following questions:
1= Sure not
5= Sure yes

	Sure not				Sure yes
**1. Do you think that XX would you lend you a large sum of money if he/she had so much?**	**1**	**2**	**3**	**4**	**5**
	a	b	c	d	e
**2. Do you think that XX would return you a loan of a large sum of money?**	**1**	**2**	**3**	**4**	**5**
	a	b	c	d	e
**3. Would you leave to XX the care of something very valuable for you?**	**1**	**2**	**3**	**4**	**5**
	a	b	c	d	e
**4. If you had a secret that could be very harmful for you if it did become public, would you feel safe to share it with XX?**	**1**	**2**	**3**	**4**	**5**
	a	b	c	d	e
**5. Do you think that XX would provide help to you in a move?**	**1**	**2**	**3**	**4**	**5**
	a	b	c	d	e
**6. If XX had to defend you hurting himself, do you think he/she would do?**	**1**	**2**	**3**	**4**	**5**
	a	b	c	d	e
